# Effect of Folic Acid Supplementation and Dietary Protein Level on Growth Performance, Serum Chemistry and Immune Response in Weanling Piglets Fed Differing Concentrations of Aflatoxin

**DOI:** 10.3390/toxins12100651

**Published:** 2020-10-09

**Authors:** Ding Wang, Merlin D. Lindemann, Mark J. Estienne

**Affiliations:** 1 Department of Animal and Food Sciences, University of Kentucky, Lexington, KY 40546, USA; dwang@miraclecorp.com; 2 Virginia Tech-Tidewater Agricultural Research and Extension Center, Suffolk, VA 23437, USA; mestienn@vt.edu

**Keywords:** aflatoxin, folic acid, protein level, piglets

## Abstract

Effects of folic acid and protein levels on growth and serum chemistry in pigs fed aflatoxin were determined in two experiments. Increasing aflatoxin (250 to 800 ppb) decreased (*P* < 0.05) weight gain and feed intake for both of the 35-day trials. In Experiment 1, increasing aflatoxin (0, 250, 500 ppb), increased linearly (*P* < 0.05) aspartate aminotransferase (AST), alkaline phosphatase (ALKP) and ɣ-glutamyl transferase (GGT). Folic acid (0, 2.0, 5.0, 12.5 ppm) increased linearly (*P* < 0.05) serum K, Ca, P, Mg, and AST with the largest effect observed at 12.5 ppm. Folic acid decreased (*P* < 0.05) blood urea nitrogen (BUN): creatinine and Na:K. In Experiment 2, aflatoxin (800 ppb) increased (*P* < 0.05) glucose and GGT, and decreased (*P* < 0.05) Na:K and albumin:globulin. Increasing protein from 15 to 18% elevated BUN: creatinine (*P* < 0.05), albumin: globulin (*P* < 0.05), albumin (*P* < 0.05) and ALKP (*P* < 0.05). Folic acid (2 ppm) elevated (*P* < 0.05) BUN, and interacted with both aflatoxin (*P* < 0.10) and protein (*P* < 0.05) on BUN. Adding folic acid to aflatoxin contaminated diets improved some measures of clinical chemistry in Experiment 1 but not traditional growth performance measures. The higher protein level reduced the effects of aflatoxicosis on growth.

## 1. Introduction 

Aflatoxins were first isolated and characterized in 1963 after 100,000 young turkeys died following the consumption of mold-contaminated peanut meal [1]. The highly toxic secondary metabolites are produced by *Aspergillus flavus, Aspergillus parasiticus*, and less commonly by other *Aspergillus* species [2]. Of the four principal aflatoxins (B_1_, B_2_, G_1_ and G_2_), aflatoxin B_1_ (AFB_1_) is the most potent and prevalent [3]. Miller [4] stated in 1995 that the Food and Agriculture Organization (FAO) estimated that 25% to 50% of the world’s food crops were affected by mycotoxins, resulting in the loss of over 1 billion tons per year of feedstuffs. More recent assessments [5] confirmed the FAO estimate of 25%, but also noted that the figure greatly underestimated the occurrence of mycotoxins above the detectable levels which may be 60–80%. In the United States, computer modeling suggests that there is a loss of USD 932,000,000 due to mycotoxin contamination, and USD 466,000,000 for regulatory enforcement, testing and other quality control measures annually [6]. Ingestion of aflatoxins by food animals such as domestic pigs (*Sus scrofa*), one of the most susceptible species, results in impaired growth and metabolism, immune suppression, and possibly death [3]. Physiological effects of aflatoxin consumption include liver damage characterized by enlargement, impaired protein synthesis, and release of enzymes into the blood such as aspartate aminotransferase (AST), ɣ-glutamyl transferase (GGT), and alkaline phosphatase (ALKP) [7]. 

Factors such as high heat and humidity stimulate colonization, growth, and toxin production by aflatoxin-producing fungi in field crops or after harvest, transportation and storage of grain. A variety of strategies have been employed, with varying success, to mitigate the effects of aflatoxins in contaminated grains such as treatment with acids or ammonia, or supplementing feed with adsorbents and binders [3]. Moreover, Combs and Harrison [8] reported that the addition of 2 ppm folic acid to finishing pig diets containing roasted corn contaminated with 550 ppb aflatoxin resulted in a 10% improvement in feed conversion efficiency. A subsequent trial conducted at the same location, also adding 2 ppm folic acid and corn contaminated with 550 ppb aflatoxin, but using younger pigs (approximately 11 kg body weight), however, demonstrated no such beneficial response to folic acid [9]. Research with bacteria [10] and guinea pigs [11] also suggested benefit in some situations of aflatoxin exposure. In further work with domestic swine, pigs fed for 49 days a diet containing approximately 840 ppb AFB_1_ displayed growth rates that were reduced to approximately 54% of that of pigs that did not receive the aflatoxin-contaminated diet [12]; the addition of 2 ppm folic acid to the aflatoxin-contaminated diet resulted in a 32% increase in growth rate, which was a 37.5% recovery of the weight gain difference between the positive and negative control pigs. These observations of occasional large responses to relatively low levels of folic acid supplementation were the impetus for our further evaluation of the relationship between folic acid supplementation and diets contaminated with aflatoxin. Moreover, an increased dietary protein or amino acid level and higher protein quality has been reported to have beneficial effects on reducing aflatoxicosis [13,14]. Aflatoxin might interfere with utilization of dietary protein by inhibiting synthesis of DNA, RNA and protein, and higher protein diets might promote the metabolism of aflatoxin by the hepatic microsomal drug-metabolizing system [15]. 

Thus, the objectives of this research were to assess the possible interactive effects of folic acid supplementation and protein level on growth performance, serum chemistry and immune response, in weaned pigs fed differing levels of aflatoxin.

## 2. Results

### 2.1. Experiment 1

#### 2.1.1. Growth Performance

Table 1 contains measures of growth performance, and means for all treatment combinations are presented. With the exceptions of F:G for the periods from d 0 to 21 and days 0 to 35 (*P* < 0.05) (described later), response measures were not affected by aflatoxin and folic acid level interactions. Thus, for clarity of presentation, the main effects of aflatoxin level and folic acid level on growth performance measures are shown in Table 2. Increasing aflatoxin ingestion linearly decreased (*P* < 0.05) body weight at days 21 and 35 as a result of reduced ADG (linear, *P* < 0.05) with increasing dietary aflatoxin level from day 0 to 21, day 22 to 35, and from day 0 to day 35. The cumulative effect over the 35-day trial period in pigs fed 500 ppb aflatoxin was a 25% reduction in ADG (Table 2). The reduction in ADG for pigs fed 250 ppb aflatoxin was 7.7% with that reduction occurring principally during the initial three weeks of the study. There was also a quadratic effect (*P* < 0.05) of aflatoxin level on ADG for the period from day 22 to 35, and tendencies for quadratic effects on body weights on day 35 and ADG from day 0 to 35. The decreases in ADG were associated with linear decreases (*P* < 0.05) in ADFI from day 0 to 21, day 22 to 35, and day 0 to 35. The F:G was not affected by increasing aflatoxin concentration during the first 21 days, but decreased with the increasing dietary aflatoxin concentration from 0 to 500 ppm during days 22 to 35 and days 0 to 35 (linear, *P* < 0.05 and quadratic, *P* < 0.05). 

There were no effects (*P* > 0.10) of folic acid supplementation on ADG or ADFI. The F:G was affected in a quadratic fashion (*P* < 0.05) for the periods from day 0 to 21, day 22 to 35, and day 0 to 35. This was accompanied by the interaction (*P* < 0.05) of folic acid supplementation and aflatoxin level during days 0 to 21 and days 0 to 35. The interaction resulted from differences among aflatoxin levels at where the apex of the F:G response curve occurred.

#### 2.1.2. Serum Clinical Chemistry

Albumin (linear, *P* < 0.05) and the ratio of albumin to globulin (linear, *P* < 0.05), but not blood urea nitrogen (BUN) or total protein were decreased with increasing aflatoxin ingestion (Table 3). The three serum enzymes (AST, ALKP and GGT) increased linearly (*P* < 0.05) with increasing aflatoxin levels. Supplementation of folic acid linearly increased K (*P* = 0.05), Ca (*P* < 0.05), P (*P* < 0.05), Mg (*P* < 0.05), total protein (tendency, *P* < 0.10), and AST (*P* = 0.05) with the largest effects observed at the 12.5 ppm supplementation level. Folic acid supplementation also decreased the ratio of Na: K (linear, *P* < 0.05).

Interactions between aflatoxin ingestion and folic acid supplementation were observed for glucose (*P* < 0.05), BUN (*P* < 0.05), creatinine (*P* < 0.05) and the BUN to creatinine ratio (BUN: Creatine (*P* < 0.05). The concentrations of glucose, BUN, and the ratio of BUN: creatinine, although within the reference range for pigs [16], were inexplicably low for the pigs fed 0 ppb aflatoxin with 12.5 ppm folic acid compared to other treatment groups which accounted for much of the interactions.

#### 2.1.3. Immune Response

The primary immune response, as determined from blood samples collected on day 21 (Table 4), indicated that serum antibody titers to ovalbumin linearly increased with increasing aflatoxin ingestion (*P* < 0.01). There were no significant effects observed for the primary immune response to concanavalin A or the secondary immune response to either ovalbumin or concanavalin A.

### 2.2. Experiment 2

#### 2.2.1. Growth Performance

Table 5 contains measures of growth performance, with means for all treatment combinations presented. The main effects of aflatoxin, protein and folic acid levels on growth are shown in Table 6. Both aflatoxin ingestion and lower dietary crude protein content negatively impacted pig body weights on days 21 and 35 (*P* < 0.01). An interaction between aflatoxin and crude protein on body weight was also detected on these days (*P* < 0.05) with a 23% reduction in body weight on day 35 in pigs consuming the high aflatoxin (800 ppb), low protein (15%) diet but only a 12% reduction when pigs consuming 800 ppb aflatoxin were fed the diet containing 18% crude protein. Pig ADG was reduced (*P* < 0.01) by either increasing dietary aflatoxin level from 0 to 800 ppb or reducing dietary crude protein level from 18 to 15% during day 0 to 21, day 22 to 35, and day 0 to 35. The ADFI was decreased (*P* < 0.01) by aflatoxin ingestion during all three periods. Main effect means (Table 6) demonstrated a 26% reduction in ADG with increasing aflatoxin ingestion for the 35-day trial, which was due to the 26% decrease in ADFI. This was accompanied by an interaction (*P* = 0.02) with protein level for ADG for the overall trial. The depression in growth rate due to aflatoxin presence in the diet was 35.6% (348 vs. 540 g) at the 15% crude protein level but only 17.1% (494 vs. 596 g) at the 18% crude protein level. There was an adverse effect of aflatoxin on F:G during the period from day 0 to 21 that was largely due to the interaction with crude protein level. No significant main effects of folic acid supplementation or interactions with either aflatoxin level or crude protein content of the diet were observed on ADG, ADFI and F:G.

#### 2.2.2. Serum Clinical Chemistry

With regard to the effects of the treatments on serum chemistry values (Table 7), the aflatoxin concentration of the diet resulted in a decrease in the albumin: globulin ratio (*P* = 0.05) and an elevation in GGT (*P* < 0.05). Aflatoxin ingestion also increased glucose level (*P* < 0.05), and decreased Na:K ratio (*P* < 0.05). Increasing protein level from 15 to 18% elevated serum BUN: creatinine ratio (*P* < 0.05), albumin: globulin ratio (*P* < 0.05), albumin (*P* < 0.05) and ALKP (*P* < 0.05) values, and tended to increase serum total protein level (*P* < 0.10) and BUN level (*P* = 0.10). The addition of folic acid to the diet at 2 ppm reduced serum glucose (*P* < 0.05) and ALKP (*P* < 0.05), and elevated (*P* < 0.05) BUN and total bilirubin. Folic acid also had interactive effects on BUN and creatinine with both aflatoxin level (*P*< 0.10) and protein level (*P* < 0.05); folic acid also had an interactive effect on ALKP levels with protein level (*P* < 0.10).

## 3. Discussion

### 3.1. Growth Performance

In agreement with the results of earlier research [7,13,17,18,19,20], increasing aflatoxin ingestion by pigs in the current study decreased body weights, ADG and ADFI. The increased F:G in pigs fed 800 ppb aflatoxin (Experiment 2) is also consistent with previous results of Lindemann et al. [12] and Schell et al. [7] which demonstrate that younger, smaller pigs are more sensitive than older, larger pigs to any given level of aflatoxin. The reductions in growth rate were the result of reductions in feed intake with no effect of aflatoxin on F:G in the initial three-week period (day 0 to 21) in Experiment 1. However, the quadratic response in F:G for the total trial (day 0 to 35), in pigs fed increasing levels of aflatoxin from 0 to 500 ppb (Experiment 1) was unexpected. That pigs fed diets containing aflatoxin appeared to be more feed efficient than pigs fed control diets is difficult to explain in biological terms and is perhaps an artifact of the large difference in body size of the pigs on the respective treatments given the understanding that heavier pigs are less efficient to a degree because of greater energetic maintenance costs which are a function of bodyweight [21]. 

The quadratic effects of folic acid supplementation from 0 to 12.5 ppm of the diet observed on F:G are very interesting, as earlier research in pigs was mainly focused on the supplemental effect of folic acid on the reproductive performance of sows [22,23,24,25]. Moreover, in Experiment 1 of the current report, there was an interaction of folic acid supplementation and aflatoxin level. The interaction resulted from differences among aflatoxin levels at where the apex of the F:G response curve occurred. This interaction was not observed in Experiment 2, when the diet was supplemented with 2 ppm folic acid. Additionally, the fact that the response curve did not plateau but returned to control values coupled with the additional fact that F:G values for the period from day 22 to 35 cannot be accurately compared due to large difference in body size of pigs on different treatments, discounts the value of this statistical response in feed conversion efficiency and suggests that it may be an anomaly.

The importance of maintaining proper protein supplementation for nursery pigs has been extensively studied with interactions with other dietary components also observed [26]. Decreasing protein level from 18% to 15% herein decreased ADG of piglets as expected; the depression in the 35-day ADG due to aflatoxin presence in the diet was 35.6% (348 vs. 540 g) at the 15% crude protein level but only 17.1% (494 vs. 596 g) at the 18% crude protein level. This result indicates that higher protein levels may, to some degree, counteract the negative effects of aflatoxin on growth in pigs. 

### 3.2. Serum Clinical Chemistry

In agreement with earlier reports [12,18], ingestion of aflatoxin increased serum levels of selected liver-specific enzymes, including serum AST, ALKP, and GGT, as well as tended to alter total bilirubin, in nursery pigs. AflatoxinB_1_ is predominantly metabolized in the liver by specific cytochrome P450 enzymes to produce an AFB_1_-8, 9-exo-epoxide [2]. This compound can then bind to proteins and cause acute toxicity (aflatoxicosis) or to DNA forming a promutagenic AFB_1_-N7-guanine DNA adduct that results in G to T transversion mutations, increasing the risk of hepatocellular cancer [2]. A potential beneficial effect of folic acid supplementation is evidenced by the decreasing serum ALKP (Experiment 2), but this hypothetical benefit was not supported by changes in the other serum components studied in this experiment. Nevertheless, at high aflatoxin levels (800 ppb), or low protein levels (15%), the supplementation of 2 ppm folic acid robustly acted to return ALKP values nearer to normal control values. Supplementation of folic acid to diets containing higher protein (18%) or without aflatoxin was without effect since ALKP values were already normal. Though not statistically significant, this same relationship was demonstrated in the near restoration of total protein and albumin values when folic acid was added to diets containing aflatoxin or suboptimal protein levels; situations which cause adverse effects on these indicators of protein synthesis.

Indeed, BUN, total protein and albumin values are all indicators of protein synthesis and typically are depressed in situations of aflatoxicosis [7]. In the present experiments, only albumin was reduced with increased aflatoxin in Experiment 1 which has been reported before [7,12,14].

Albumin and globulins, the two major constituents of serum proteins, play a pivotal role in the inflammatory process [27]. The decrease in serum albumin and albumin: globulin ratio was consistent with the increase in the three selected liver-specific enzymes, which indicate reduced protein synthesis and the damage of the liver with chronic inflammation and reflects a cumulative exposure of various proinflammatory cytokines [27]. The reduction in albumin: globulin ratio at different dietary protein levels was consistent with the reduced protein intake. 

The reduction in BUN and BUN: creatinine ratio and the tendency of creatinine to be linearly increased with aflatoxin ingestion may be related to renal damage because high creatinine levels indicate poor kidney function. The decrease in serum Na: K ratio, which has been reported to be an index for renal function [28], is consistent with potential renal damage. A low BUN: creatinine ratio may also be linked to a diet low in protein and a severe muscle injury called rhabdomyolysis [29]. The interaction between aflatoxin ingestion and folic acid supplementation on BUN and creatinine is worthy of further research.

### 3.3. Immune Response

Immunosuppression caused by aflatoxin B_1_ has been demonstrated in various livestock species [3,30,31,32], however the suppression in cellular immunity was more easily demonstrated than suppressed humoral immunity [17]. The linear increase in serum antibody titers to ovalbumin indicated that primary immunity in pigs was increased, rather than decreased, by aflatoxin ingestion. The lack of primary and secondary immune responses after treatment with concanavalin A in association with aflatoxin was previously reported [33]. van Heugten et al. [17] reported that the viability of unstimulated mononuclear cells after 66 h culture was greater for pigs fed diets containing 280 ppb of aflatoxin, and no differences were detected in stimulation indices for concanavalin A and purified protein derivatives when control diets were compared with diets containing either 400 or 800 ppb of aflatoxin. 

## 4. Conclusions

Aflatoxicosis was observed in pigs in this study and reducing the dietary crude protein level from 18% to 15% exacerbated the aflatoxicosis. There were, however, no improvements in traditional growth response parameters due to folic acid supplementation. The effects observed herein would have presumably been more pronounced if the study were continued for additional weeks, since the aflatoxin effects would have been compounded with time and that may affect the practical field responses to aflatoxicosis.

## 5. Materials and Methods

### 5.1. General

While the experiments were conducted at Virginia Tech at a time and in a place that was not under formal institutional experimental review the studies were, nevertheless, conducted in accordance with the Guide for the Care and Use of Agricultural Animals in Research and Teaching [34], the document whereby institutions evaluate experimental conduct. Yorkshire x Chester White x Duroc pigs, weaned at 26 to 32 days of age and allowed a 7 to 10-day post-weaning adjustment period, were used in two experiments conducted to evaluate the effects of dietary folic acid supplementation and protein level on growth performance, serum chemistry and immune response under situations of differing levels of aflatoxin ingestion.

Pigs were housed in pens of three pigs each (in Experiment 1, two pens had only two pigs each due to the available number of pigs). Pens measured 0.9 × 1.2 m and were situated over plastic coated woven wire in an environmentally controlled nursery room. Temperature and ventilation were adjusted weekly based on pig size and age. Feed and water were available on an ad libitum basis. Pigs were weighed at the start of the trial (day 0) and at days 21 and 35 (end of trial). When pigs were weighed, feed disappearance was recorded to allow for determination of average daily feed intake (ADFI) and the ratio of feed to gain (F:G). Average daily gain (ADG), ADFI, and F:G were calculated for the periods from day 0 to 21, 22 to 35, and 0 to 35. 

### 5.2. Blood Sampling and Analyses

For both experiments, blood samples were collected via jugular venipuncture on day 35. Serum was harvested following centrifugation (3000× g for 15 min at 4 °C), and stored at −20 °C until analyzed. Alterations in serum chemistry were used as indicators of changes in liver function due to treatment. Serum analyses were performed at the Virginia-Maryland College of Veterinary Medicine (Blacksburg, VA, USA) using a Kodak Ektachem 700 analyzer (Eastman Kodak, Rochester, NY, USA). 

For Experiment 1 only, all pigs were given a 1 mL intramuscular injection of an antigenic mixture containing ovalbumin (3 mg/mL; A5376, Sigma Chemical Co., St. Louis, MO, USA) and concanavalin A (C2631, Sigma Chemical Co.) on day 0 to allow for assessment of treatment effects on humoral immunity. Blood samples were obtained from the anterior vena cava on day 21 of the study to assess the primary immune response. Pigs were then re-injected with the antigenic mixture and blood was sampled again on day 35 for assessment of the secondary immune response. Serum antibody titers to ovalbumin and concanavalin A were determined using a passive hemagglutination assay described by Barnette et al. [35]. In the antibody assay, ovalbumin and concanavalin A were attached to sheep red blood cells via chromic chloride coating. Serial dilutions of serum were made to determine the antibody titers.

### 5.3. Experiment 1

A total of 106 pigs (60 castrate males, 48 females) weighing 9.73 ± 0.76 kg (mean ± SE) were blocked according to sex and body weight and then randomly assigned within each block to one of the 12 dietary treatment combinations for a 3 × 4 factorial arrangement. There were three levels of aflatoxin inclusion (0, 250, and 500 ppb) and four levels of folic acid supplementation (0, 2.0, 5.0, and 12.5 ppm), with three replications per treatment combination. 

The control diet (0 ppb aflatoxin; Table 8, column 1) was a corn and soybean meal-based diet formulated to 1.00% lysine and fortified with vitamins and minerals to meet or exceed NRC [36] requirement estimates. Soybean oil was added at a rate of 1% for dust suppression. Due to limited availability of naturally contaminated corn, the decision was made to utilize a source of aflatoxin developed from a culture of *Aspergillus parasiticus* on rice starch using the procedure of Shotwell et al. [37] with the modifications of West et al. [38] and Wiseman et al. [39]. The aflatoxin content was determined by the spectrophotometric analysis described by Nabney and Nesbitt [40] as modified by Wiseman et al. [39]. The mixed aflatoxin obtained (88% of aflatoxin was AFB_1_) was added to the control diet in appropriate amounts to achieve the desired aflatoxin levels (250 or 500 ppb). A pilot study with the aflatoxin source indicated that its utilization at the rate of 400 ppb resulted in performance effects similar to those previously observed in our facilities when approximately 800 ppb aflatoxin from naturally contaminated grain was used. The different folic acid levels were achieved by using a folate premix (2000 mg folic acid per kg corn) that replaced corn at appropriate inclusion levels.

### 5.4. Experiment 2

A total of 72 pigs (40 castrate males, 32 females) weighing 9.95 ± 0.56 kg, were blocked by sex and body weight and randomly allotted within each block to one of the eight treatment combinations for a 2 × 2 × 2 factorial arrangement. There were two levels of aflatoxin (0 and 800 ppb), two protein levels (15 and 18%) and two folic acid supplementation levels (0 and 2 ppm), with three replicates per treatment combination. 

The experimental diets are shown in Table 1. Grain naturally contaminated with aflatoxin was obtained and utilized. The aflatoxin contamination level (800 ppb) was achieved by using the appropriate blend of contaminated corn (2305 ppb AFB_1_) and uncontaminated corn (0 ppb AFB_1_). The aflatoxin content of the corns utilized in this experiment was determined by a high-performance liquid chromatography procedure [41] at the Virginia-Maryland College of Veterinary Medicine. Dietary protein level differences were achieved by altering the amount of corn and soybean meal which resulted in differences in the dietary lysine content; mineral supplements were altered to maintain dietary Ca and P content. The different folic acid levels were achieved by using a folate premix (2000 mg folic acid per kg corn) that replaced corn at the appropriate inclusion level. 

### 5.5. Statistical Analyses

Data were analyzed using the MIXED procedures of SAS (SAS Institute, Inc., Cary, NC, USA) for a completely randomized design. For growth performance, the pen was considered as the experimental unit while for serum chemistry and immunity responses, each pig was considered as the experimental unit. The model included dietary treatment (aflatoxin, folic acid, protein level and their interactions) as fixed effects and block as random effects. Coefficients for unequally spaced contrasts were generated by PROC IML of SAS to evaluate polynomial treatment responses for Experiment 1. Least squares means are reported and an alpha level of 0.05 was used for the declaration of statistical significance and 0.10 for the declaration of a tendency.

## Figures and Tables

**Table 1:** Effects of aflatoxin ingestion and folic acid supplementation levels on growth performance of pigs (Experiment 1).

Aflatoxin, ppb:	0				250				500				SEM
Folic Acid, ppm:	0	2	5	12.5	0	2	5	12.5	0	2	5	12.5	
Body weight, kg													
Day 0	9.67	9.77	9.61	9.80	9.70	9.92	9.81	9.70	9.67	9.64	9.66	9.84	0.76
Day 21 ^a^	19.86	19.80	20.09	20.29	18.77	18.79	18.88	18.38	17.45	17.98	17.11	17.58	1.42
Day 35 ^ac^	29.64	28.84	29.91	29.33	27.97	28.24	27.62	28.10	24.75	24.97	23.26	24.90	1.78
Average Daily Gain, g													
Day 0–21 ^a^	486	478	499	499	432	423	432	413	370	397	355	368	34
Day 22–35 ^ab^	698	646	702	646	657	674	624	694	521	499	439	523	42
Day 0–35 ^ac^	571	545	580	558	522	523	509	526	431	438	389	430	33
Average Daily Feed Intake, g													
Day 0–21 ^a^	943	971	931	986	816	852	874	806	720	751	763	720	66
Day 22–35 ^a^	1634	1879	1764	1631	1393	1517	1426	1447	1141	1138	1037	1188	104
Day 0–35 ^a^	1220	1334	1264	1244	1047	1118	1094	1063	888	906	873	907	76
Feed:Gain													
Day 0–21 ^de^	1.94	2.03	1.86	1.97	1.89	2.02	2.03	1.95	1.94	1.90	2.15	1.95	0.04
Day 22–35 ^abd^	2.34	2.91	2.50	2.54	2.12	2.25	2.28	2.09	2.20	2.30	2.36	2.28	0.11
Day 0–35 ^abde^	2.14	2.45	2.17	2.23	2.01	2.14	2.15	2.02	2.06	2.08	2.24	2.11	0.05

Note: LSMeans are presented with each mean representing three pens of pigs. ^a^ Linear effect of aflatoxin, *P* < 0.05; ^b^ Quadratic effect of aflatoxin, *P* < 0.05; ^c^ Quadratic effect of aflatoxin, 0.05 < *P* < 0.10; ^d^ Quadratic effect of folic acid, *P* < 0.05; ^e^ Interaction between aflatoxin and folic acid, *P* < 0.05.

**Table 2:** Main effects of aflatoxin ingestion and folic acid supplementation levels on growth performance of pigs (Experiment 1).

	Aflatoxin, ppb			Folic Acid, ppm			
	0	250	500	0	2	5	12.5
Body Weight, kg							
Day 0	9.71	9.78	9.70	9.68	9.78	9.69	9.78
Day 21 ^a^	20.01	18.71	17.53	18.69	18.86	18.70	18.75
Day 35 ^ac^	29.43	27.98	24.47	27.45	27.35	26.93	27.44
Average Daily Gain, g							
Day 0–21 ^a^	490	425	373	429	432	429	427
Day 22–35 ^ab^	673	662	496	626	606	588	621
Day 0–35 ^ac^	563	520	422	508	502	492	505
Average Daily Feed Intake, g							
Day 0–21 ^a^	958	837	739	826	858	856	838
Day 22–35 ^a^	1727	1446	1126	1390	1511	1409	1422
Day 0–35 ^a^	1266	1081	894	1052	1119	1077	1071
Feed:Gain							
Day 0–21 ^de^	1.95	1.97	1.99	1.93	1.98	2.01	1.96
Day 22–35 ^abd^	2.58	2.19	2.29	2.22	2.49	2.38	2.30
Day 0–35 ^abde^	2.25	2.08	2.13	2.07	2.22	2.19	2.12

Note: LSMeans are presented with each main effect mean for aflatoxin level representing twelve pens of pigs and each main effect mean for folic acid level representing nine pens of pigs. ^a^ Linear effect of aflatoxin, *P* < 0.05; ^b^ Quadratic effect of aflatoxin, *P* < 0.05; ^c^ Quadratic effect of aflatoxin, 0.05 < *P* < 0.10; ^d^ Quadratic effect of folic acid, *P* < 0.05; ^e^ Interaction between aflatoxin and folic acid, *P* < 0.05.

**Table 3:** Effects of aflatoxin ingestion and folic acid supplementation levels on serum chemistry of pigs on day 35 (Experiment 1).

Aflatoxin, ppb:	0				250				500				SE
Folic Acid, ppm:	0	2	5	12.5	0	2	5	12.5	0	2	5	12.5	
Glucose ^af^, mg/dL	120.8	107.6	119.4	96.9	124.2	124.8	123.2	120.8	118.6	109.4	112.4	126.1	5.6
BUN ^f^, mg/dL	11.7	10.3	13.7	7.9	11.5	12.9	10.9	12.4	12.2	10.2	9.0	12.2	1.0
CRT ^bf^, mg/dL	0.85	0.90	0.97	0.87	0.89	0.96	0.94	0.97	0.93	0.91	0.89	1.07	0.04
BUN:CRT ^cf^	13.71	11.29	14.47	9.17	13.01	13.25	11.52	12.83	13.16	11.09	9.87	11.74	1.11
Na ^g^, mmol/L	145.9	148.8	149.9	145.2	147.0	148.5	149.4	149.3	147.9	146.9	147.9	151.9	1.7
K ^c^, mmol/L	6.58	6.60	6.38	6.98	6.61	6.65	7.14	6.95	6.55	6.63	7.28	6.91	0.26
Na:K ^c^	22.58	22.80	23.64	20.88	22.53	22.60	21.09	21.56	22.73	22.41	20.61	22.07	0.73
CL, mmol/L	101.8	105.1	105.0	101.1	102.5	103.6	104.8	103.7	103.2	101.9	103.1	106.3	1.5
CO_2_, mmol/L	27.0	26.9	28.1	28.0	26.6	28.1	26.8	28.2	28.0	28.0	27.9	27.0	0.6
Anion gap, mmol/L	23.7	23.7	23.0	23.1	24.5	23.5	25.3	24.1	23.3	23.6	24.2	25.4	0.9
Ca ^bcg^, mg/dL	10.00	10.50	10.74	10.25	10.78	10.93	10.71	11.11	10.57	10.30	10.47	11.14	0.21
Mg ^cf^, mg/dL	1.99	2.18	2.03	2.09	2.13	1.95	2.02	2.18	1.86	2.07	2.02	2.24	0.06
P ^c^, mg/dL	8.44	9.10	8.93	9.32	9.10	9.15	9.37	9.43	8.86	8.68	8.77	9.09	0.28
TP ^dg^, g/dL	6.04	6.71	6.52	6.43	6.41	6.46	6.16	6.66	6.08	6.11	6.24	6.89	0.19
ALB ^ad^, g/dL	3.97	4.30	4.19	4.18	4.16	4.05	3.88	4.20	3.70	3.67	3.56	4.21	0.17
ALB:GLB ^ae^	1.98	1.81	1.84	1.89	1.88	1.70	1.79	1.74	1.60	1.52	1.33	1.59	0.11
AST ^ac^, U/L	50.4	56.4	48.7	63.1	51.9	54.0	57.3	57.9	56.3	62.2	70.2	62.7	4.6
ALKP ^a^, U/L	195.0	188.3	169.1	176.7	215.0	214.6	264.4	246.1	255.1	249.3	320.1	257.2	29.7
GGT ^a^, U/L	42.2	41.1	40.1	44.6	46.9	45.4	58.0	44.8	61.4	71.7	65.0	59.4	7.5
Total bili ^b^, mg/dL	0.11	0.15	0.10	0.20	0.15	0.14	0.17	0.17	0.13	0.23	0.22	0.16	0.04

Note: LSMeans are presented with each mean representing eight or nine pigs. BUN, blood urea nitrogen; CRT, creatinine; TP, total protein; ALB, albumin; ALB:GLB, albumin:globulin; AST, aspartate aminotransferase; ALKP, alkaline phosphatase; GGT, gamma glutamyl transferase; Total bili, total bilirubin. ^a^ Linear effect of aflatoxin, *P* < 0.05; ^b^ Linear effect of aflatoxin, 0.05 < *P* < 0.10; ^c^ Linear effect of folic acid, *P* < 0.05; ^d^ Linear effect of folic acid, 0.05 < *P* < 0.10; ^e^ Quadratic effect of folic acid, 0.05 < *P* < 0.10; ^f^ Interaction between aflatoxin and folic acid, *P* < 0.05; ^g^ Interaction between aflatoxin and folic acid, 0.05 < *P* < 0.10.

**Table 4:** Effects of aflatoxin ingestion and folic acid supplementation levels on primary and secondary humoral immune response of pigs on days 21 and 35 (Experiment 1).

Aflatoxin ppb:	0				250				500				SEM
Folic Acid, ppm:	0	2	5	12.5	0	2	5	12.5	0	2	5	12.5	
CONA I	2.4	2.4	2.1	2.6	2.0	3.1	1.6	2.8	1.7	2.1	2.7	1.9	0.4
CONA II	4.0	3.5	3.0	4.2	3.8	4.4	2.8	4.5	2.7	3.3	3.7	3.4	0.6
OVAL I ^a^	0.7	1.1	1.0	0.9	1.6	1.1	0.8	1.1	1.0	2.4	1.7	2.1	0.5
OVAL II	5.6	5.5	6.3	5.5	5.4	6.8	4.8	5.6	5.5	5.9	6.6	5.8	1.1

Note: LSMeans are presented with each mean representing eight or nine pigs. CONA I and II = primary (day 21) and secondary (day 35) response, respectively, to concanavalin A; OVAL I and II = primary and secondary response, respectively, to ovalbumin. Each positive serial dilution was assigned a value of 1, and the sum for each pig was used for the analysis. ^a^ Linear effect of aflatoxin, *P* < 0.05.

**Table 5:** Effects of aflatoxin ingestion, dietary crude protein, and folic acid supplementation levels on growth performance of pigs (Experiment 2).

Aflatoxin, ppb:	0				800				SEM
Crude Protein, %:	15		18		15		18		
Folic Acid, ppm:	0	2	0	2	0	2	0	2	
Body Weight, kg									
Day 0	9.99	9.93	10.03	9.90	9.89	9.94	9.90	9.97	0.56
Day 21 ^abc^	20.48	20.25	22.21	21.04	16.48	16.33	19.10	19.51	1.02
Day 35 ^abc^	28.53	26.60	29.72	29.33	21.29	21.14	25.75	26.32	1.70
Average Daily Gain, g									
Day 0–21 ^abd^	499	492	580	530	314	304	438	455	29
Day 22–35 ^abe^	697	558	609	754	449	421	576	587	64
Day 0–35 ^abc^	568	511	596	596	352	343	486	501	25
Average Daily Feed Intake, g									
Day 0–21 ^a^	1152	1214	1140	1051	919	818	850	919	84
Day 22–35 ^a^	1778	1703	1674	1714	1217	1048	1267	1375	116
Day 0–35 ^a^	1376	1388	1332	1283	1012	899	999	1072	87
Feed:Gain									
Day 0–21 ^bcf^	2.31	2.47	1.95	1.99	2.95	2.69	1.94	2.02	0.15
Day 22–35 ^gh^	2.56	3.09	2.83	2.37	2.73	2.50	2.20	2.33	0.22
Day 0–35 ^b^	2.42	2.72	2.23	2.15	2.86	2.62	2.06	2.13	0.11

Note: LSMeans are presented with each mean representing three pens of pigs. ^a^ Aflatoxin effect, *P* < 0.01; ^b^ Protein effect, *P* < 0.01; ^c^ Interaction between aflatoxin and protein level, *P* < 0.05; ^d^ Interaction between aflatoxin and protein level, 0.05 < *P* < 0.10; ^e^ Interaction between protein level and folic acid, 0.05 < *P* < 0.10; ^f^ Aflatoxin effect, *P* < 0.05; ^g^ Aflatoxin effect, 0.05 < *P* < 0.10; ^h^ Protein effect, 0.05 < *P* < 0.10.

**Table 6:** Main effects of aflatoxin ingestion, dietary crude protein, and folic acid supplementation levels on growth performance of pigs (Experiment 2).

Level	Aflatoxin, ppb		Crude Protein, %		Folic Acid, ppm	
	0	800	15	18	0	2
Body Weight, kg						
Day 0	9.96	9.93	9.94	9.95	9.96	9.94
Day 21 ^abc^	20.99	17.86	18.38	20.46	19.57	19.28
Day 35 ^abc^	28.54	23.63	24.39	27.78	26.32	25.85
Average Daily Gain, g						
Day 0–21 ^abd^	525	378	402	501	458	445
Day 22–35 ^abe^	654	508	531	632	583	580
Day 0–35 ^abc^	568	421	444	545	501	488
Average Daily Feed Intake, g						
Day 0–21 ^a^	1139	877	1026	990	1015	1001
Day 22–35 ^a^	1717	1227	1436	1508	1484	1460
Day 0–35 ^a^	1345	995	1169	1171	1179	1161
Feed:Gain						
Day 0–21 ^bcf^	2.18	2.40	2.60	1.98	2.29	2.29
Day 22–35 ^gh^	2.71	2.44	2.72	2.43	2.58	2.57
Day 0–35 ^b^	2.38	2.42	2.65	2.14	2.39	2.41

Note: LSMeans are presented with each main effect mean for each treatment factor representing twelve pens of pigs. ^a^ Aflatoxin effect, *P* < 0.01; ^b^ Protein effect, *P* < 0.01; ^c^ Interaction between aflatoxin and protein level, *P* < 0.05; ^d^ Interaction between aflatoxin and protein level, 0.05 < *P* < 0.10; ^e^ Interaction between protein level and folic acid, 0.05 < *P* < 0.10; ^f^ Aflatoxin effect, *P* < 0.05; ^g^ Aflatoxin effect, 0.05 < *P* < 0.10; ^h^ Protein effect, 0.05 < *P* < 0.10.

**Table 7:** Effects of aflatoxin, protein level, and folic acid supplementation on serum chemistry values of pigs on day 35 (Experiment 2).

Aflatoxin, ppb	0				800				SEM
Crude Protein, %	15		18		15		18		
**Folic Acid, ppm**	0	2	0	2	0	2	0	2	
Glucose ^ab^, mg/dL	103.0	86.3	102.5	94.0	103.8	102.5	116.8	98.7	6.6
BUN ^bcde^, mg/dL	7.8	12.8	11.8	13.8	9.5	12.2	12.1	9.8	1.3
CRT ^de^, mg/dL	0.77	1.05	0.85	0.88	0.92	0.92	0.93	0.78	0.10
BUN:CRT ^f^	10.22	11.77	14.10	15.98	10.78	13.60	14.26	12.97	1.35
Na, mmol/L	143.7	138.2	142.7	142.3	145.2	143.7	140.7	141.7	4.4
K ^g^, mmol/L	5.76	5.51	5.57	5.77	6.24	6.43	5.89	5.93	0.35
Na:K ^a^	25.07	25.77	25.82	24.88	23.50	22.84	24.20	23.98	1.07
CL, mmol/L	100.3	96.4	99.3	100.0	103.2	102.1	97.0	99.3	3.8
CO_2_, mmol/L	26.8	25.4	24.2	26.7	24.9	25.7	27.0	25.5	1.1
Anion gap, mmol/L	22.2	22.3	25.2	22.0	23.7	22.2	22.8	23.2	0.8
Ca, mg/dL	9.37	9.46	9.78	9.60	9.57	9.76	9.65	9.80	0.61
Mg, mg/dL	1.63	1.64	1.83	1.68	1.58	1.68	1.65	1.75	0.22
P, mg/dL	7.48	7.89	8.12	7.92	7.42	8.05	7.53	7.78	0.57
TP ^c^, g/dL	5.55	5.63	5.85	5.80	5.02	5.73	5.77	6.02	0.47
ALB ^f^, g/dL	3.37	3.34	3.72	3.63	2.78	3.30	3.53	3.57	0.33
ALB:GLB ^af^	1.54	1.46	1.75	1.68	1.25	1.37	1.58	1.45	0.08
AST, U/L	46.8	56.0	51.0	51.0	50.3	54.9	52.8	55.3	6.3
ALKP ^bfh^, U/L	300.7	174.3	166.3	182.0	321.8	226.9	201.2	179.2	37.0
GGT ^a^, U/L	43.8	57.0	43.3	37.0	77.3	62.2	67.0	84.0	8.2
Total bili ^b^, mg/dL	0.10	0.17	0.10	0.15	0.12	0.16	0.13	0.13	0.03

Note: LSMeans are presented with each mean representing nine pigs. BUN, blood urea nitrogen; CRT, creatinine; TP, total protein; ALB, albumin; ALB:GLB, albumin:globulin; AST, aspartate aminotransferase; ALKP, alkaline phosphatase; GGT, gamma glutamyl transferase; Total bili, total bilirubin. ^a^ Aflatoxin effect, *P* < 0.05; ^b^ Folic acid effect, *P* < 0.05; ^c^ Protein effect, 0.05 < *P* < 0.10; ^d^ Interaction between protein level and folic acid, *P* < 0.05; ^e^ Interaction between aflatoxin and folic acid, 0.05 < *P* < 0.10; ^f^ Protein effect, *P* < 0.05; ^g^ Aflatoxin effect, 0.05 < *P* < 0.10; ^h^ Interaction between protein and folic acid, 0.05 < *P* < 0.10.

**Table 8:** Composition (g/kg) of diets for the experiments ^a^.

Aflatoxin, ppb:	0		800	
Crude Protein, %:	18	15	18	15
Ingredient				
Clean corn	687	774.6	340	427.6
Contaminated corn ^b^	--	--	347	347
Soybean meal, 48%	269	180	269	180
Soybean oil	10	10	10	10
Di-calcium phosphate	12	13.8	12	13.8
Limestone	10	9.6	10	9.6
Vitamin premix ^c^	2.5	2.5	2.5	2.5
Trace mineral premix ^d^	0.5	0.5	0.5	0.5
Salt	4	4	4	4
ASP-250 ^e^	5	5	5	5
	1000	1000	1000	1000
Nutrients, calculated %				
Crude protein	18.9	15.3	18.9	15.3
Lysine	1.01	0.76	1.01	0.76
Calcium	0.76	0.76	0.76	0.76
Phosphorus	0.59	0.59	0.59	0.59
Meth + cys	0.65	0.56	0.65	0.56

^a^ For Experiment 1, the diet in the first column (0 ppb Aflatoxin and 18% crude protein) was used as the basal diet to which appropriate amounts of a cultured source of aflatoxin were added. In Experiment 2, all four diets were used. The different folic acid (No. 101725, ICN Pharmaceuticals, Inc., Costa Mesa, CA, USA) supplementation levels were achieved by using a special folate premix (2000 mg folic acid per kg of corn) that was prepared and that replaced corn at the appropriate inclusion level. ^b^ Contaminated corn contained 2300 ppb aflatoxin B_1_. ^c^ Supplied per kilogram of diet: 4.34 mg of riboflavin, 22 mg of pantothenic acid, 22 mg of niacin, 22 µg of vitamin B_12_, 440 mg of choline chloride, 4409 IU of vitamin A, 441 IU of vitamin D_3_, 11 IU of vitamin E, 1102 µg of vitamin K (as menadione sodium bisulfate complex), and 0.3 mg of Se. ^d^ Supplied per kilogram of diet: 150 mg of Zn, 176 mg of Fe, 60 mg of Mn, 17 mg of Cu, 2 mg of I. ^e^ ASP-250 (Zoetis, Parsippany, NJ, USA) contains per kg: 44 g chlortetracycline, 22 g procaine penicillin and 4.4% sulfamethazine.
